# Abnormal expression of miRNA-122 in cerebral infarction and related mechanism of regulating vascular endothelial cell proliferation and apoptosis by targeting CCNG1

**DOI:** 10.1016/j.clinsp.2023.100199

**Published:** 2023-04-27

**Authors:** Xiao-Juan Yu, Tian Zhang, Zeng-Zhen Wei, Bin Gu, Ting Guo, Wen-Juan Jiang, Yue-Qin Shen, Dong Wang, Qian Wang, Jun Wang

**Affiliations:** a Emergency Department, Taizhou People's Hospital Affiliated to Nanjing Medical University, Taizhou, P.R. China; b Clinical Laboratory, Taizhou People's Hospital Affiliated to Nanjing Medical University, Taizhou, P.R. China; c Blood Purification Center, Taizhou People's Hospital Affiliated to Nanjing Medical University, Taizhou, P.R. China

**Keywords:** miRNA-122, Acute cerebral infarction, CCNG1, Apoptosis, Vascular endothelial cell

## Abstract

**Objective:**

To analyze the value of serum miRNA-122 expression in the diagnosis, severity, and prognosis of Acute Cerebral Infarction (ACI) and the correlation mechanism of serum miRNA-122 on the proliferation and apoptosis of vascular endothelial cells in ACI.

**Method:**

A total of 60 patients with ACI who were admitted to the emergency department of the Taizhou People's Hospital from January 1, 2019, to December 30, 2019, and 30 healthy controls during the same period were selected. General clinical data of all patients at admission were collected. Including age, sex, medical history, and inflammatory factors (C-Reactive Protein [CRP], Interleukin-6 [IL-6], Procalcitonin [PCT], Neutrophil Gelatinase-Associated Lipid carrier protein [NGAL]). The National Institutes of Health Stroke Scale (NIHSS) score at admission and short-term prognosis (the Modified Rankin Score [mRS]) score at 3 months after onset were recorded. The expression level of miRNA-122 in the serum of patients with ACI and normal controls was detected by reverse-transcription quantitative Real-Time Polymerase Chain Reaction (RT-QPCR), and the correlation between the expression level of miRNA-122 in the serum of patients with ACI and the level of inflammatory factors, NIHSS and mRS scores were analyzed. The expression levels of miRNA-122 in the serum of patients with ACI, normal people, and Human Umbilical cord Endothelial Cells (HUVECs) cultured in a blank control group were detected by RT-QPCR and statistically analyzed. MTT and flow cytometry was used to compare the proliferation and apoptosis of vascular endothelial cells in the miRNA-122 mimics and inhibitors transfection groups and the corresponding negative control group. The mRNA and protein levels of apoptosis-related factors Bax, Bcl-2, Caspase-3, and angiogenesis-related proteins Hes1, Notch1, Vascular Endothelial Growth Factors (VEGF), and CCNG1 were detected by RT-QPCR and Western blot. Bioinformatics methods predicted CCNG1 to be the target of miRNA-122, and the direct targeting relationship between CCNG1 and miRNA-122 was verified by a dual-luciferase reporting assay.

**Result:**

Serum miRNA-122 expression in patients with ACI was significantly higher than that in healthy controls, with an area under the receiver operating characteristic curve of 0.929, 95% Confidence Interval of 0.875‒0.983, and an optimal cut-off value of 1.397. The expression levels of CRP, IL-6, and NGAL in patients with ACI were higher than those in healthy control groups, p < 0.05; miRNA-122 was positively correlated with CPR, IL-6, NIHSS score, and mRS score. At 48h and 72h, the proliferation rate of HUVECs cells in the miRNA-122 mimics group decreased and the apoptosis rate increased. Cell proliferation rate increased, and apoptosis rate decreased significantly in the groups transfected with miRNA-122 inhibitors. The mRNA and protein levels of pro-apoptotic factors Bax and caspase-3 were significantly increased in the miRNA-122 mimics transfection group, while those of anti-apoptotic factor Bcl-2 were significantly decreased compared to those of the control group. The expression of Bax and Caspase-3 decreased, and the expression of anti-apoptotic factor Bcl-2 increased in the transfected miRNA-122 inhibitors group. mRNA expression levels of Hes1, Notch1, VEGF, and CCNG1 in the miRNA-122 mimic transfected group were significantly decreased, while mRNA expression levels in the miRNA-122 inhibitors transfected group were significantly increased. Bioinformatics showed that there was a miRNA-122 binding site in the 3′UTR region of CCNG1, and dual luciferase assay confirmed that CCNG1 was the target of miRNA-122.

**Conclusion:**

Serum miRNA-122 increased significantly after ACI, which may be a diagnostic marker of ACI. miRNA-122 may be involved in the pathological process of ACI and is related to the degree of neurological impairment and short-term prognosis in patients with ACI. miRNA-122 may play a regulatory role in ACI by inhibiting cell proliferation, increasing apoptosis, and inhibiting vascular endothelial cell regeneration through the CCNG1 channel.

## Highlights

• miRNA-122 in cerebral infarction.
• miRNA-122 may be involved in the pathological process of ACI.
• miRNA-122 related to the degree of neurological impairment and short-term prognosis in patients with ACI.

## Introduction

Acute Cerebral Infarction (ACI) is a common cerebrovascular occlusive disease seen in an emergency department.^1^ ACI is also a leading cause of disability and death.^2^ In the treatment of ACI, saving cerebral tissue in an ischemic penumbra region is the key to treatment. Previous studies have found that the prognosis of patients with cerebral infarction is related to neovascularization in the infarct area.^2^ The formation of neovascularization is based on the proliferation, migration, and reconstruction of vascular endothelial cells. miRNA is a small non-coding RNA that plays an important role in the physiological and pathological processes of many diseases.^3^ miRNA is closely related to the pathological process of ACI as well as to many risk factors.^4^, ^5^, ^6^, ^7^ In the study of ACI, Cheng et al. found that up-regulation of miRNA-122 can alleviate stroke after middle cerebral artery occlusion in rats, suggesting that miRNA-122 is closely related to the course of ACI, but clinical studies are limited.^8^ In addition, a growing number of studies have shown that ACI can induce the release of pro-inflammatory cytokines, which can induce inflammatory responses and further promote the progression of ACI. An excessive inflammatory response after cerebral ischemia and hypoxia is one of the important mechanisms leading to reperfusion injury, which requires the infiltration of inflammatory cells and the participation of some cytokines, intercellular adhesion molecules, and chemokines.^9^^,^^10^ There is a correlation between ACI and inflammatory factors and miRNA. As a Cell Cycle regulator, Cyclin G1 (CCNG1), is mainly involved in the G2 phase and G2/M phase transformation regulation.^11^ Previous studies related to tumors have confirmed that CCNG1 is the target of Mir-23b through luciferase activity reporting test, and Mir-23b inhibits lung cancer cell proliferation by directly targeting CCNG1.^12^ Chang et al. found that INcRNA LINC 01494 promoted glioma proliferation, migration, and invasion through miRNA-122-5P/CCNG 1 axis.^13^ CCNG1 also plays an important role in regulating the vascular endothelial cell cycle. This study aimed to explore the clinical value of miRNA-122 in patients with ACI and the related mechanism of regulating the proliferation and apoptosis of vascular endothelial cells by targeting CCNG1, to provide a new perspective for the diagnosis and treatment of cerebral infarction.

## Material and methods

### Patients

#### Clinical sample collection

**Fig. 1:**
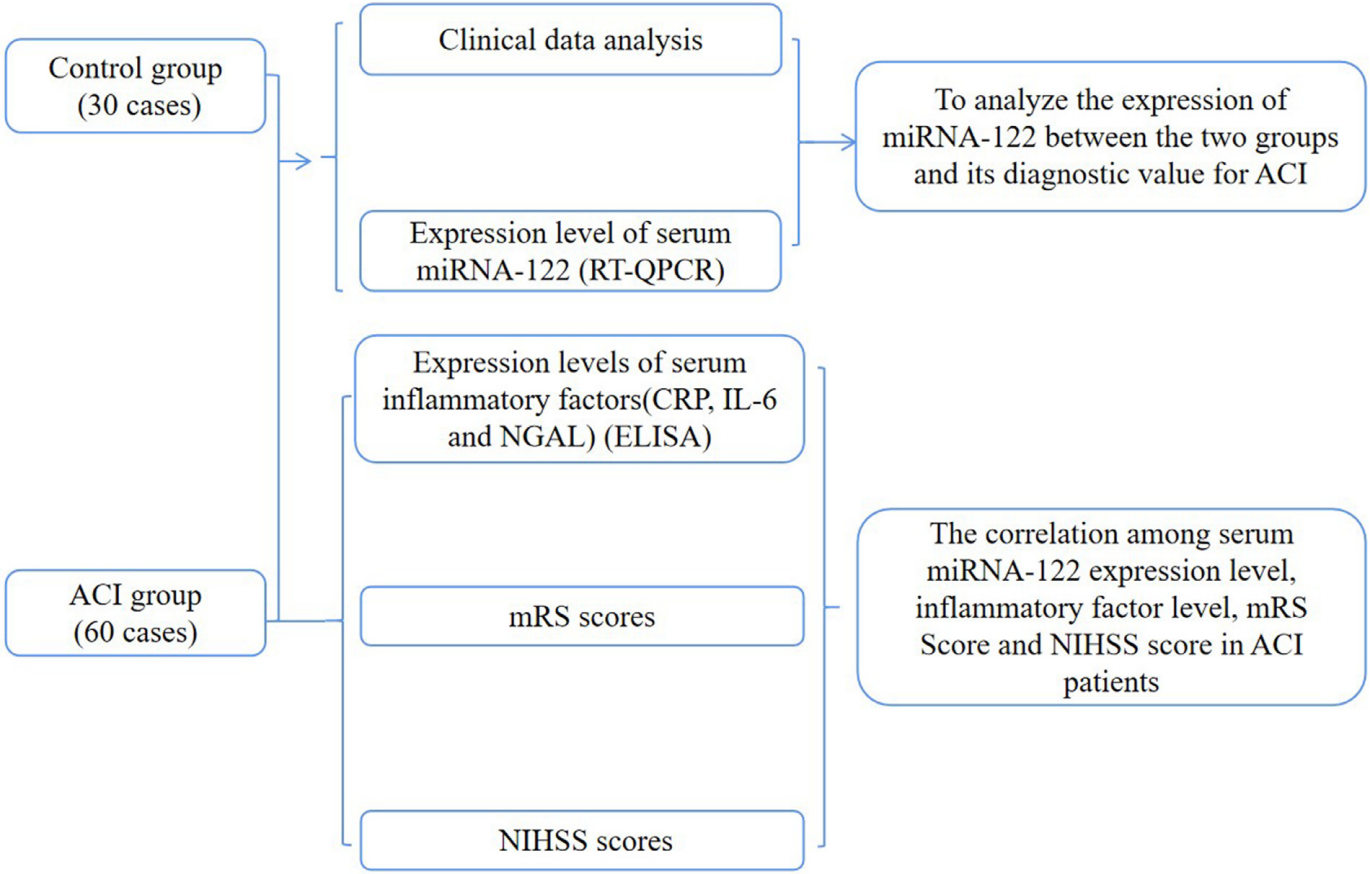
The flow diagram for the clinical study section.

Serum samples from 60 patients with ACI and 30 healthy participants who were diagnosed in the Emergency Department of the Taizhou People's Hospital from January 2020 to December 2021 were included in this study. General clinical data such as age, gender, hypertension, diabetes, coronary heart disease, history of atrial fibrillation, history of hyperlipidemia, smoking, and drinking were collected. The patient's neurological functions were assessed on admission and the National Institutes of Health Stroke Scale (NIHSS) score was calculated. Patients’ prognoses were assessed according to the modified Rankin Score (mRS) 3-months after a stroke, and follow-up was performed. The expression levels of miRNA-122 and values of inflammatory factors C-Reactive Protein (CRP), IL-6, and Neutrophil Gelatinase-Associated Lipid carrier protein (NGAL) were recorded at admission. All patients were new and signed informed consent forms. The Ethics Committee of Taizhou People's Hospital approved the study (approval nº KY 201813701), and all samples were treated anonymously in accordance with ethical and legal standards. The research was following the STROBE. The flow diagram is shown in Fig. 1.

#### Inflammatory factor detection

Serum inflammation was detected by Enzyme-Linked Immunosorbent Assay (ELISA) to detect serum CRP, IL-6, and NGAL levels. The samples to be tested and different concentrations of the standards were added to a well, pre-coated with the relevant capture antibody. Incubated at 37°C for 1h, washed five times, the substrate was then added and incubated at 37 (PM) for 15 min. Finally, the stop buffer was added and mixed properly, and the absorbance was determined. Each experiment was repeated thrice strictly following the instructions on the kit.

#### Cell culture

The Human Umbilical Vein Cells (HUVECs) (purchased from the Shanghai Institute of Cell Biology) were cultured in vitro. The cells of the control group were cultured in M199 medium containing 5% serum (Gibco, USA) and 1% cyanin-streptomycin (Gibco, USA) in an incubator at 37°C and 5% CO_2_ (Thermo, USA). Logarithmic growth phase cells were selected for subsequent experiments.

#### Real-time quantitative polymerase chain reaction

Trizol was used to extract RNA from cells and then synthesize cDNA. Polymerase Chain Reaction (PCR) was carried out in strict accordance with kit instructions (Thermo, USA). To calculate multiple changes in gene expression, 2^−ΔΔCt^ method was used. Each Real-Time Quantitative Polymerase Chain Reaction (RT-QPCR) was set with three replicates. Specific primers for these genes were designed and synthesized by Sangon Biotechnology Co., LTD. (Shanghai, China). Primer sequences were as follows: miRNA-122 (Forward: CTCAACTGGTGTCGTGGAGTCGGCAATTCAGTTGAGCAAACACC; Reverse: TCGCCTGGAGTGTGACAATGG), PCR reaction conditions: pre-denaturation at 95°C, 10 min; Denaturation at 95°C for 15s, then annealing at 60°C for 60s, 40 cycles.

#### Cell transfection

The miRNA-122 mimics (UGGAGUGUGACAAUGGUGUUUG) and miRNA-122 inhibitors (CAAACACCAUUGUCACACUCCA) were designed and synthesized by Nanjing Manforit Biotechnology Co., LTD. According to the procedure of the Lipofectamine 2000 experiment, 0.8 µg/well required miRNA-122 mimics, miRNA-122 inhibitors, a blank control, and were transfected into HUVECs cells.

#### Cell counting kit -8(CCK-8) test

HUVECs cells were inoculated in 96-well plates at a density of 1×105 cells/well for 12h, 24h, 36h, 48h, or 72h. After incubation, 10 Μl CCK-8 reagent was added (Beyotime, Guangzhou, China). The plates were then incubated for 1h. The absorbance at 490 nm was measured by Mindray, Shenzhen, China.

#### Flow cytometry FCM

HUVECs cells were trypsinized and washed twice with pre-cooled Phosphate-Buffered Saline (PBS). After centrifugation, the PBS was removed, and the cells were suspended with a density of 1×10^6^ cells/mL and labeled with 5 μL Annexin VFITC and 5 μL Propidium Iodide (PI) (BD, Annexin V-FITC/PI Apoptosis Detection Kit) for double fluorescence. Add to the suspension and incubate in the dark for 15 min. To analyze the cell cycle distribution, cells were collected and immobilized overnight in 70% ethanol at 4°C, washed with PBS, and stained with 100 μL PI staining solution (5 μg/mL Rnase A, 20 μg/mL PI in PBS) at room temperature for 30 min in the dark. It was then stained with FACSCalibur. Results were analyzed using Flowjo software (Version 10.6.2 Flowjo Inc., San Diego, California, USA).

#### Western blotting

After transfection of miRNA-122 mimic and miRNA-122 inhibitor groups, the total protein of HUVECs cells was extracted by adding RIPA buffer (Vazyme Biotech, China). Protein samples were separated by SDS-PAGE and transferred to polyvinylidene fluoride membrane by polyacrylamide sulfate gel electrophoresis. After the transfer, the membrane was sealed with 5% Bowene Serum Albumin at room temperature for 1h and subsequently incubated overnight with the required primary antibody at 4°C. The next day, the membrane was incubated with Sangon Biotech (China, 1:500 dilution) for 1h, and the protein was observed by enhanced chemiluminescence (Vazyme Biotech, China). G:BOX Chemi XX9 imaging system (Syngene, UK) for protein visualization was used. Individual western blotting was performed with primary antibody, Bax, Bcl-2, Caspase-3 antibody (Abcam, USA, 1:1000 diluted), Hes1, Notch1, Vascular Endothelial Growth Factors [VEGF], CCNG1 and GAPDH antibody (Sangon Biotech, China, 1:500 diluted).

#### Verification of dual luciferase reports

Starbase (http://starb ase.sysu.edu.cn/) was used to predict CCNG1 as the target of miRNA-122. CCNG1 Wild-Type (WT) and CCNG1 Mutant-Type (MUT) were constructed by dual fluorescein, and CCNG1 WT and CCNG1 MUT vectors were co-transfected into Mir-122 mimics or mir-122 mimics control cells and incubated for 24h. After incubation, the cells were lysed on a photometer to detect luciferase activity.

### Statistical analysis

All statistical analysis was performed using SPSS 17.0 software. Data were presented as the means ± standard deviation, and the paired Student *t*-test was performed for comparison between the two groups. The accuracy of miRNA-122 in the diagnosis of ACI was calculated by the Area Under the Curve (AUC) of the Receiver Operating Characteristic curve (ROC curve). Pearson or Spearman linear correlation analysis was used. SPSS 26.0 was used for statistical analysis, and GraphPad Prism 8 was used to draw relevant images; p < 0.05 indicated a statistically significant difference.

## Results

### Clinical data analysis

The comparison of age, gender, cerebrovascular disease risk factors, and other general clinical data between patients with ACI and the physical examination control group are shown in Table 1, with no statistical significance (p > 0.05).

**Table 1:** Comparison of clinical data between patients with ACI and the physical examination control group.

	ACI group (60 cases)	Control group (30 cases)	t/χ^2^	p
**Age**	64.21 ± 5.66	62.10 ± 6.76	1.567	0.121
**Male**	34 (56.7)	18 (60.0)	0.091	0.763
**Hypertension**	39 (65.0)	17 (56.7)	0.591	0.442
**Diabetes**	26 (43.3)	9 (40.0)	1.496	0.221
**Coronary heart disease**	9 (15.0)	3 (10.0)	0.433	0.742
**Atrial fibrillation**	14 (21.5)	5 (16.7)	0.534	0.465
**Smoking**	24 (40.0)	11 (36.7)	0.094	0.760
**Drinking**	21 (35.0)	9 (30.0)	0.225	0.635
**Hyperlipemia**	31 (51.7)	13 (43.3)	0.556	0.456

Age (years); Male, hypertension, diabetes, coronary heart disease, atrial fibrillation, smoking and drinking history (Cases [%]).

### Expression level of serum miRNA-122 and its diagnostic value in ACI

The relative expression levels of miRNA-122 in the serum of patients (ACI group) and the normal group were detected by RT-QPCR (2^−ΔΔCT^).

**Fig. 2:**
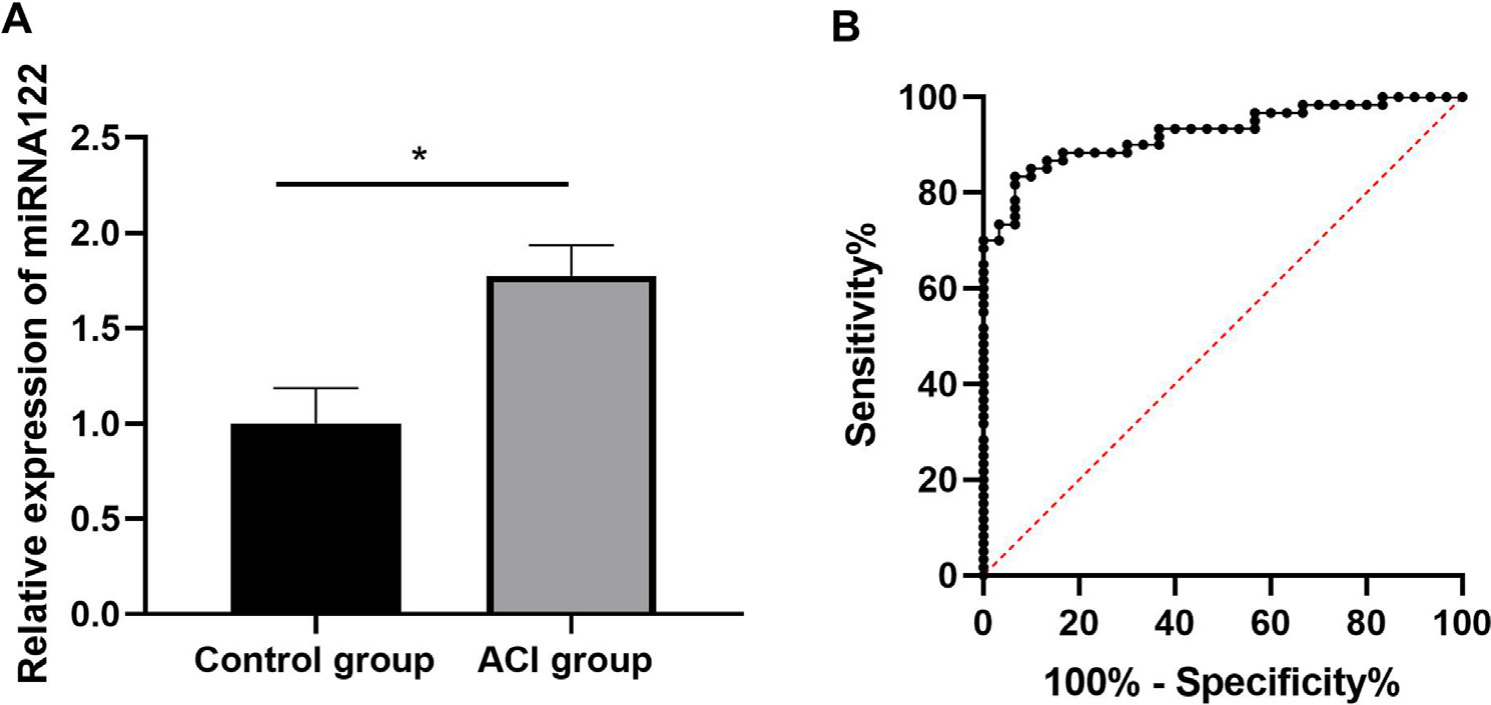
Comparison on serum miRNA-122 between group health examination and group patients with ACI (A) and the diagnostic value of miRNA-122 levels in patients with ACI; *p < 0.05 (B).

**Fig. 3:**
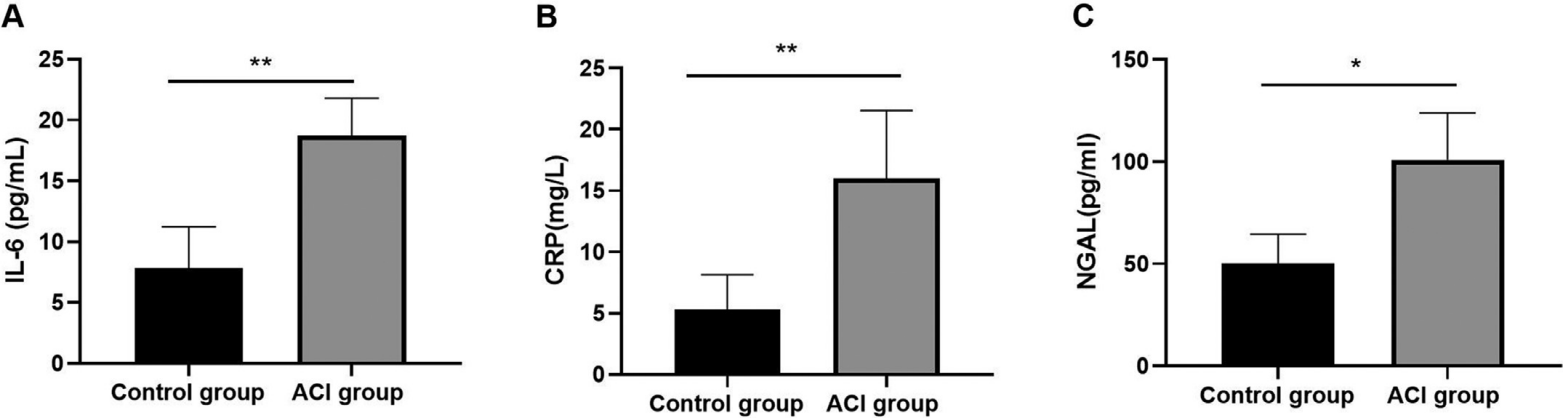
Comparison of expression levels of inflammatory factors (A: IL-6, B: CRP, C: NGAL) in each group control (control group) and acute cerebral infarction group (ACI group). (* P < 0.05, ** P < 0.01).

Fig. 2 shows that the expression levels of miRNA-122 in the ACI group were significantly up-regulated compared with that in the physical examination control group. The relative expression levels of miRNA-122 in the serum of the normal and ACI groups were (0.972 ± 0.338) and (1.842 ± 0.505), respectively, and the difference was statistically significant (p < 0.05). The AUC of the ROC curve was 0.929, as shown in Fig. 3. The 95% Confidence Interval was 0.875‒0.983, the sensitivity was 85.0%, the specificity was 90.0%, and the optimal cut-off value was 1.397. The larger the AUC, the better the diagnostic value of this marker for the disease, and the data suggest that miRNA-122 has a better diagnostic value in patients with ACI.

### Expression levels of serum inflammatory factors in patients with ACI

Inflammation occurs after ACI, and serum inflammatory factors can reflect the condition of patients with cerebral infarction. The results of this experiment showed that the expressions of CRP, IL-6 and NGAL in the serum of patients with ACI were significantly increased compared with those of the normal control group (p < 0.05), as shown in Fig. 3.

### Correlation of serum miRNA-122 expression level with CPR, IL-6, and NGAL level and short-term prognosis in patients with ACI

**Fig. 4:**
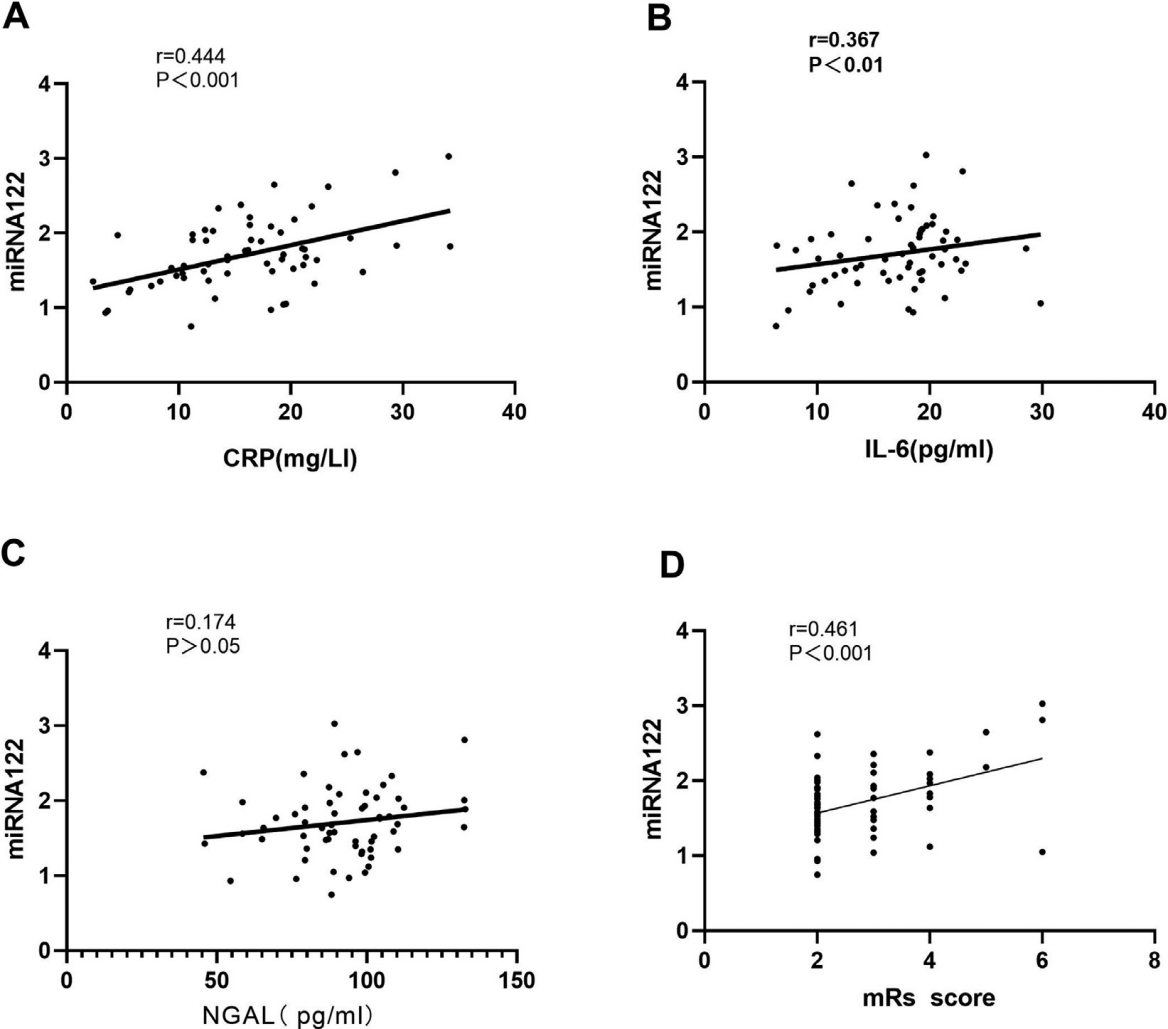
Correlation analysis of miRNA-122 and CRP (A), IL-6 (B), and NGAL (C) in the serum of patients with ACI. Correlation between the serum expression levels of miRNA-122 and the mRS score in patients with ACI (D).

To explore the relationship between miRNA-122 expression level and inflammatory factors in the serum of patients with ACI, correlation analysis results showed that miRNA-122 and CPR level, R = 0.444, p < 0.001; miRNA-122 and IL-6 levels, r = 0.367 p < 0.01; miRNA-122 and NGAL levels, r = 0.174, p > 0.05, as shown in Fig. 4. The prognostic results of mRS evaluation showed that the expression level of miRNA-122 in the serum of patients with ACI was positively correlated with mRS score (R = 0.461, p < 0.05). It is suggested that high serum miRNA-122 expression in patients with ACI has a worse prognosis with a higher mRS score. The expression level of miRNA-122 in patients may be used to evaluate their prognosis.

### Correlation between the expression levels of inflammatory factors CRP, IL-6, NGAL and mRS and NIHSS scores in patients with ACI

**Fig. 5:**
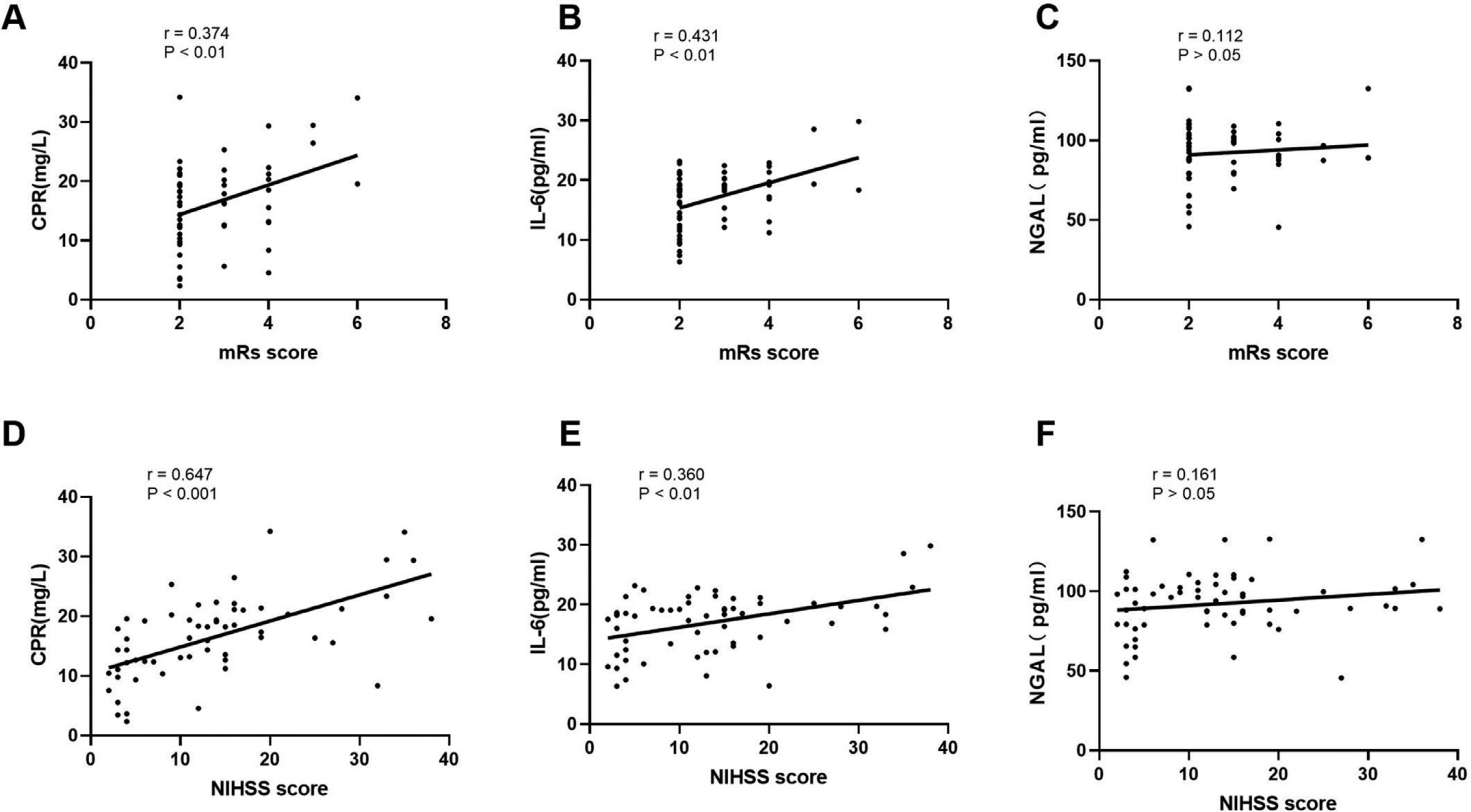
(A‒F) Association analysis of CRP, IL-6, NGAL, mRS, and NIHSS in the sera of patients with ACI.

Stroke severity was assessed by the NIHSS score, and the prognosis was assessed by the mRS 3 months after the stroke. In this study, the expressions of factors CRP, IL-6, and NGAL in the serum of patients with ACI increased, which can be used as an auxiliary diagnosis of ACI. In exploring the correlation between the prognosis of patients and assessing the severity of the stroke, the results showed that the expression levels of CRP and IL-6 in the serum of patients with ACI were positively correlated with mRS score (R = 0.374, p < 0.01) (R = 0.431, p < 0.01), but there was no correlation between NGAL expression level and mRS score (R = 0.112, p > 0.05). The expression levels of CPR and IL-6 were positively correlated with the NIHSS score (r = 0.647, p < 0.001) (r = 0.360, p < 0.01), while the expression level of NGAL was not correlated with the NIHSS score (r = 0.161, p > 0.05), as shown in Fig. 5. These results indicate that the expression levels of CPR and IL-6 are correlated with the prognosis and clinical severity of patients with ACI, although the correlation was weak, while NGAL had no correlation.

### Expression levels of miRNA-122 in serum-treated HUVECs from patients with ACI

**Fig. 6:**
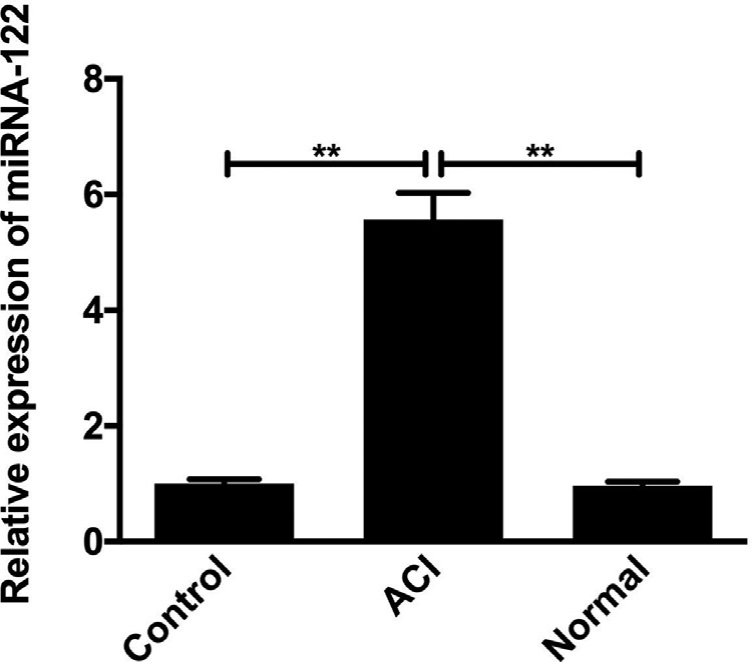
Comparison of miRNA-122 expression levels in HUVECs cells from ACI serum culture groups, normal human serum cultures, and blank control groups (**p < 0.01).

The expression levels of miRNA-122 in the serum of patients with ACI, normal human serum and HUVECs cells cultured in a blank control group were detected by RT-QPCR, as shown in Fig. 6. The results showed that the expression level of miRNA-122 in the serum culture group of patients with ACI was significantly increased.

### Effects of miRNA-122 expression on proliferation, cycle, and apoptosis of HUVECs cells

**Fig. 7:**
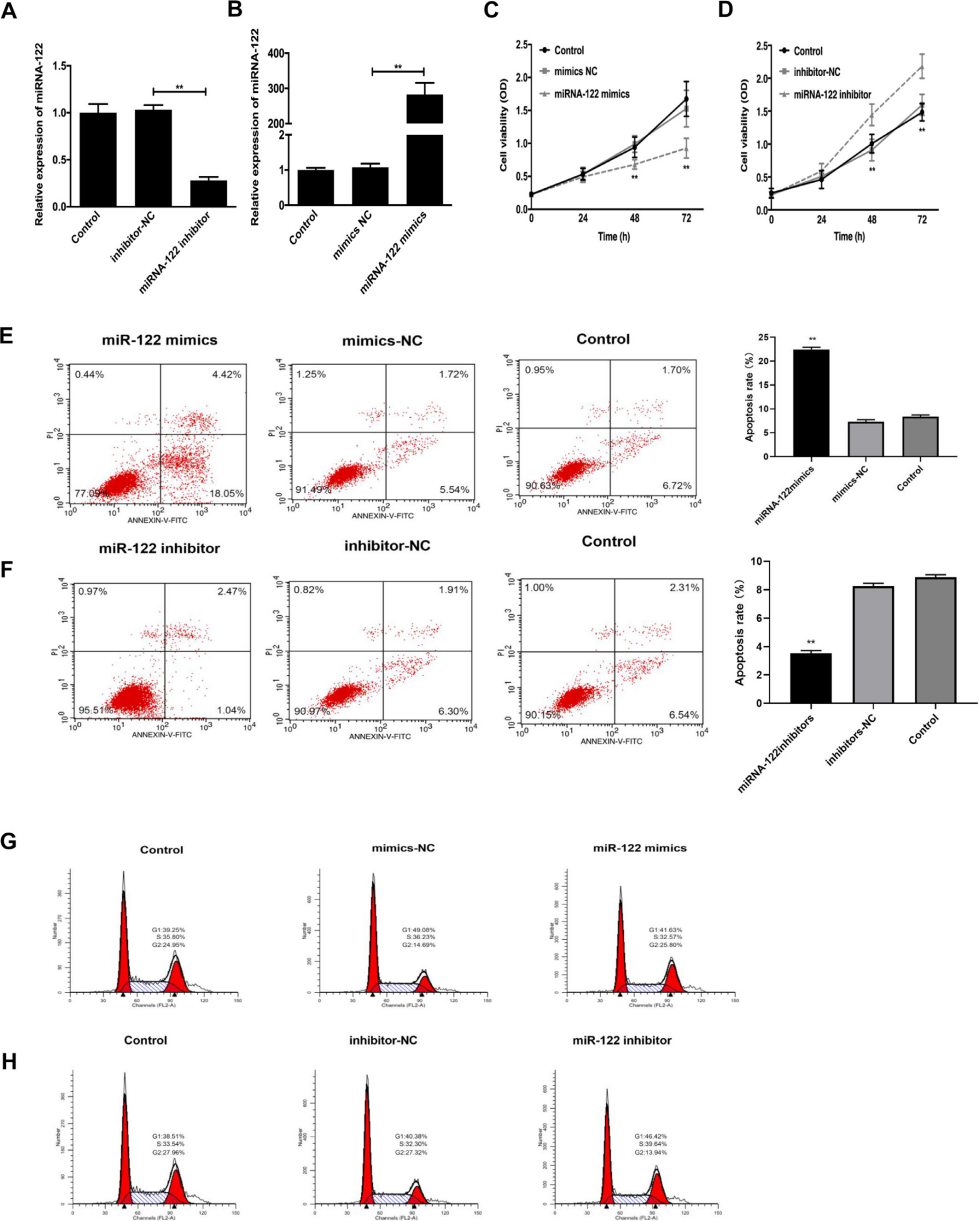
Effects of miRNA-122 expression on proliferation, cycle, and apoptosis of HUVECs cells. (A‒B) Validation of the miRNA-122 mimics/miRNA-122 inhibitors transfection efficiency; (C‒D) Performance of the cell proliferation rate in the MTT detection of HUVECs cells in the miRNA-122 mimics group and miRNA-122 inhibitors transfection group Absorbance was measured at 490 nm every 24 hours for a total of 72 hours (**p < 0.01); (E‒F) Cell flow detection Cell apoptosis rate in miRNA-122 mimics transfection, mimics NC and Control groups (**p < 0.01); (G‒H) Cell flow detection Comparison on Cell-cycle assays in different groups (**p < 0.01).

To study the effects of miRNA-122 expression on the proliferation and apoptosis of HUVECs cells, miRNA-122 mimic and miRNA-122 inhibitor models are established in Fig. 7 (A‒B). MTT cell proliferation assay showed that the proliferation rate of the miRNA-122 mimics group decreased at 48h and 72h, while that of the miRNA-122 inhibitors group increased (p < 0.01) Fig. 7 (C‒D). Flow cytometry analysis showed that the apoptosis rate of the transfected miRNA-122 mimic group was significantly increased, and the cell stagnation in the S phase was decreased, compared to those of the negative control and the blank groups (p < 0.01). The apoptosis rate in the miRNA-122 inhibitor group decreased significantly, and the cell stagnation in the S phase increased (p < 0.01), as shown in Fig. 7 (E‒H). It is concluded that miRNA-122 can regulate ACI by acting on vascular endothelial cells, that is, it can inhibit the proliferation of HUVECs cells and promote apoptosis.

### Effects of miRNA-122 on apoptosis factors of HUVECs

**Fig. 8:**
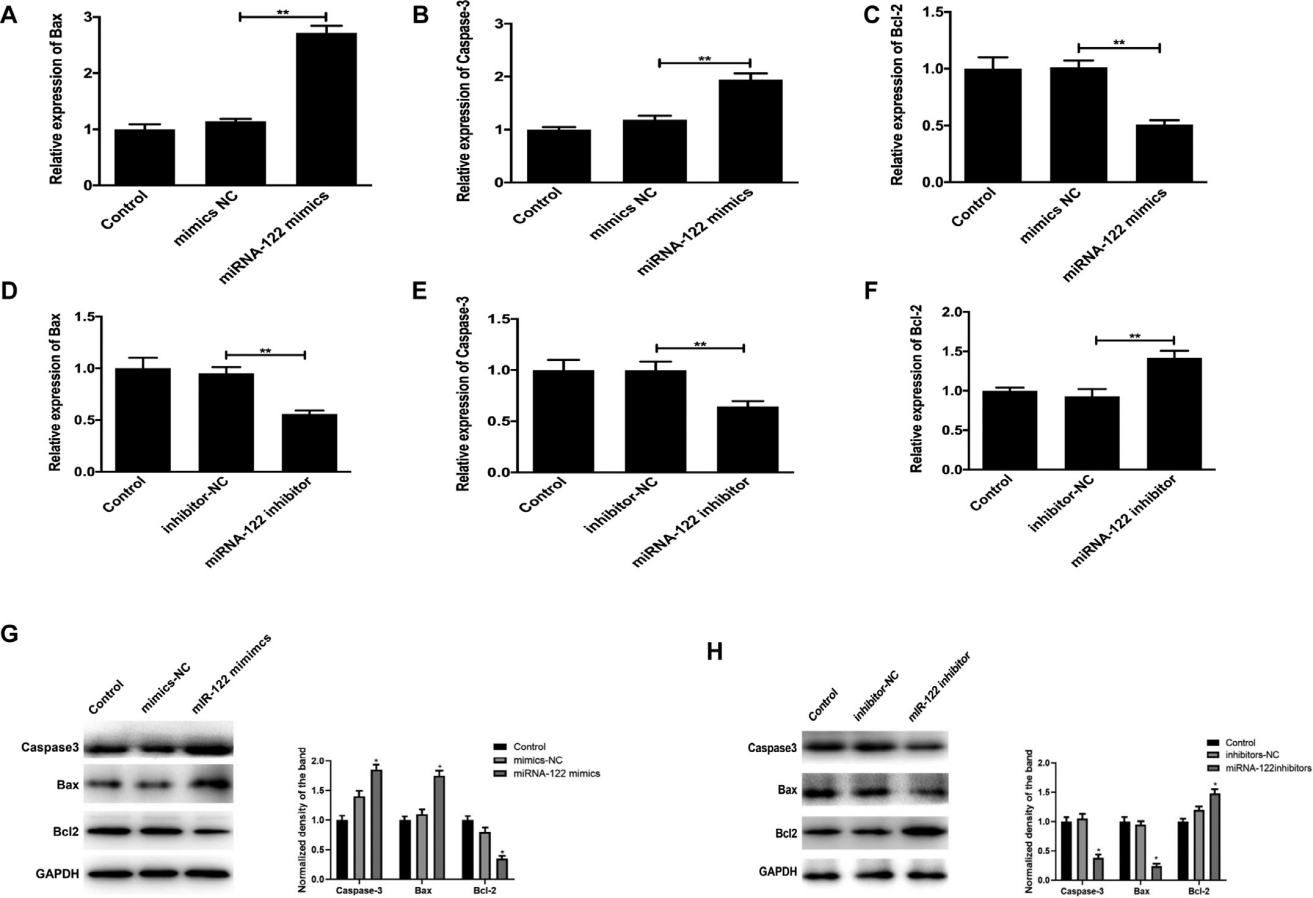
The mRNA and protein expressions of Bax, Caspase-3, and Bcl-2 in each group. (* P < 0.05, ** P < 0.01). (A‒C) Comparison on the mRNA expression levels of Bax, Caspase-3 and Bcl-2 in control, mimics-NCand miRNA-122 mimics group. (D‒F) Comparison on the mRNA expression level of Bax,Caspase-3 and Bcl-2 in control,inhibitor-NCand miRNA-122 inhibitor group. (G‒H) Comparison on the protein expression level of Bax, Caspase-3 and Bcl-2 in control, mimics-NCand miRNA-122 mimics group.

The mRNA and protein expressions of Bax, Caspase-3, and Bcl-2 in each group were detected by RT-QPCR and Western blot. The results show that compared with the mRNA and protein expressions of the control group, mRNA and protein expressions of pro-apoptotic factors Bax and Caspase-3 were significantly increased in the miRNA-122 mimic group, while anti-apoptotic factor Bcl-2 was significantly decreased. In the miRNA-122 inhibitor group, the expressions of pro-apoptotic factors Bax and Caspase-3 were significantly decreased, while the anti-apoptotic factors Bcl-2 were significantly increased, with statistical significance (p < 0.01), as shown in Fig. 8.

### Changes in mRNA and protein levels of angiogenesis related proteins in HUVECs cells by miRNA-122

**Fig. 9:**
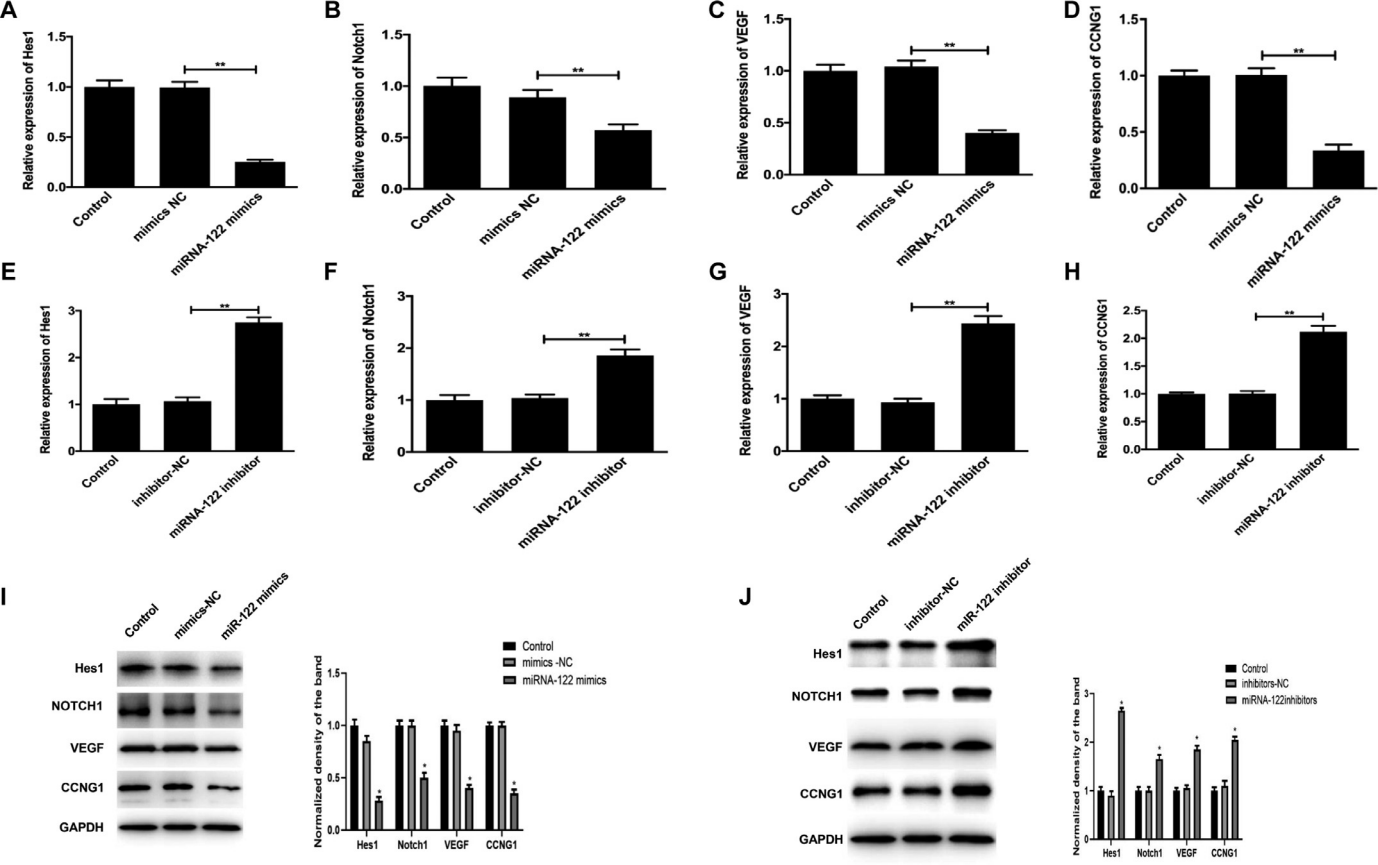
Changes in mRNA and protein levels of angiogenesis-related proteins in HUVECs cells by miRNA-122. (A‒H) Comparison on the mRNA level expression of Hes1, Notch1, VEGF, and CCNG1 in the various groups, p < 0.01; (I‒J) Protein expression levels and quantification comparisons (p < 0.05).

**Fig. 10:**
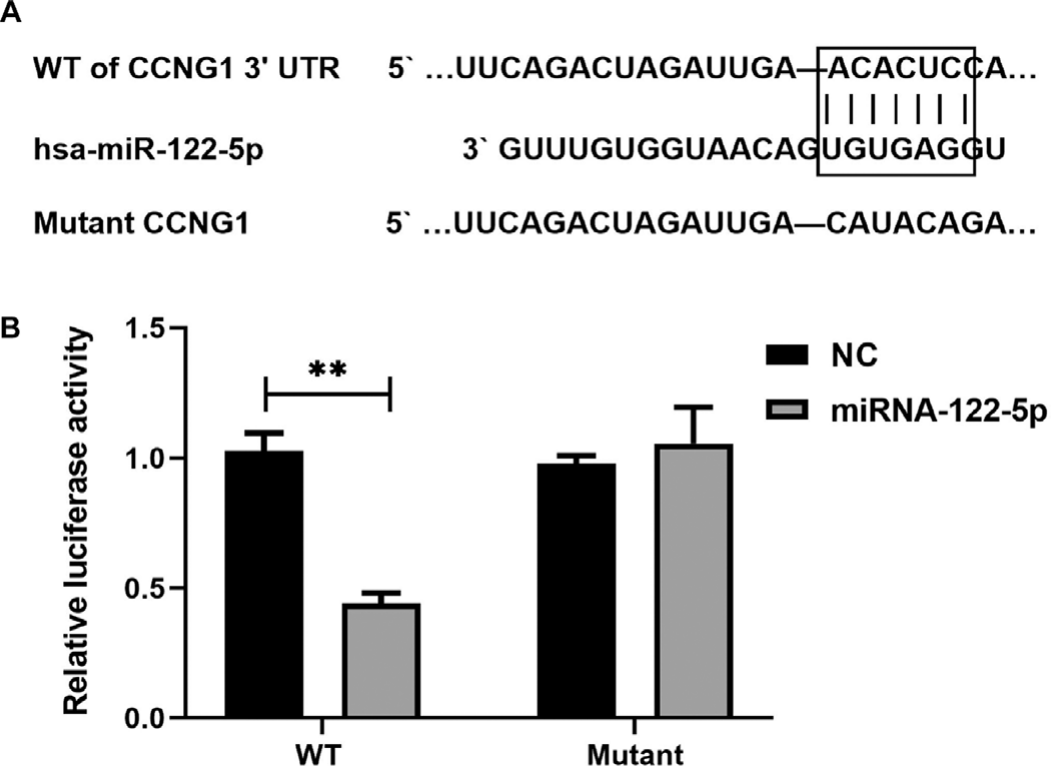
CCNG1 is a direct target gene of miRNA-122. (A) The 3’UTR segment of CCNG has a binding site for miRNA-122. (B) The miRNA-122 mimic activity significantly inhibited the luciferase activity of the Psicheck-2-X-3UTR (wild-type).

Detection results of the expressions of angiogenesis-related proteins Hes1, Notch1, VEGF, and CCNG1 showed that compared with the mRNA and protein expressions of the control group, the mRNA and protein expressions of Hes1, Notch1, VEGF, and CCNG1 in the miRNA-122 mimic group were significantly decreased (p < 0.01), while the miRNA-122 inhibitor group was significantly increased, as shown in Fig. 9. These results suggest that miRNA-122 may play a role in HUVECs cells by regulating angiogenic proteins Hes1, Notch1, VEGF, and CCNG1. Meanwhile, the results indicate that miRNA-122 may play a role in the regulation of ACI by inhibiting vascular endothelial cell proliferation and angiogenesis (Fig. 10).

### Target prediction of miRNA-122

Bioinformatics showed that there was a binding site of miRNA-122 in the 3′UTR region of CCNG1. To confirm that miRNA-122 affects the expression level of targeted CCNG1 by acting on the 3′UTR region of CCNG1, a dual-luciferase reporting system was designed. The results showed that the expression level of miRNA-122 was higher, resulting in a 2-fold decrease in luciferase activity in the 3′UTR region of CCNG1 WT (p < 0.001) than that of the control group, while there was no significant change in luciferase activity in the 3′UTR region of mutant CCNG1 (p > 0.05).

## Discussion

ACI is a common and frequently occurring disease in the clinical neurology department, with high disability and fatality rates.^14^ To date, the diagnosis of ACI mainly depends on imaging. It is crucial to find new serum markers of ACI from the perspective of serology for its diagnosis, treatment, and prognosis assessment. miRNA-122 plays an important role in the pathological process of the liver, cardiovascular, tumor, infection, and other diseases.^15^, ^16^, ^17^ The expression of miRNA-122 was significantly increased after ACI in animal models.^18^ In this study, it was further found that the expression of miRNA-122 was significantly increased in the peripheral blood of patients with ACI, and it can become a diagnostic marker for ACI.

In recent years, more studies have found that ACI can trigger the release of inflammatory factors and induce the occurrence of inflammatory responses.^19^^,^^20^ Inflammatory reaction runs through the whole pathological process after the occurrence of ACI.^21^ The higher risk of infection after ACI is associated with the suppression of immune function caused by cerebral infarction.^22^ However, the immune suppression of the body is conducive to the occurrence of inflammatory response. Conversely, the inflammatory response further worsens the dysfunction of the nervous system.^23^ The prognosis of patients with ACI is not only related to the degree of nervous system damage but also the main cause of the poor prognosis of ACI.^24^ After an ACI, inflammatory factors are activated and rapidly released, increasing their concentration in the brain tissue and surrounding blood.^25^ The release of inflammatory factors and response promotes the development of apoptosis.^26^

Previous studies have shown that miRNA-122 plays an important role in the pathogenesis of inflammation, lipid regulation, and cell apoptosis.^27^, ^28^, ^29^ In this study, it was found that the expression levels of CRP, IL-6, and NGAL in the serum of patients with ACI increased, suggesting that ACI is closely related to inflammation. Previous studies have also found that CRP expression is elevated within 3h of the onset of ACI.^30^ IL-6 expression is increased after ACI and can be used as a predictor of stroke-associated infection.^31^ NGAL gained considerable diagnostic and prognostic values in kidney disorders as a valuable marker of renal injury.^32^ Interestingly, however, this study showed that NGAL expression was elevated in the serum of patients with cerebral infarction. Previous studies have also reported that the expression level of NGAL increased after ACI.^33^ It is suggested that NGAL can not only be used in the evaluation of renal function but also reflect the inflammatory response of the body after cerebral infarction.^34^ This study also found that CRP was correlated with NIHSS score upon admission, and CRP and IL-6 levels could determine prognosis and disease severity. Previous studies have also found that CRP and IL-6 are correlated with the TOAST classification of ACI.^35^

After ACI, angiogenesis is combined with neurogenesis, that is, ischemic neural progenitor cells can promote angiogenesis by secreting VEGF in vitro.^36^ miRNA can regulate angiogenesis. For example, the down-regulation of Mir-195 by targeting VEGF A can promote angiogenesis after cerebral infarction.^37^ Compared with the control group, overexpression of miRNA-122 significantly reduced the proliferation rate of HUVECs at 48h and 72h and increased the apoptosis rate, and decreased cell stagnation at the S phase of the cell cycle. However, transfection with miRNA-122 inhibitor significantly increased cell proliferation and reduced cell apoptosis rate. These results suggest that miRNA-122 can inhibit the proliferation of vascular endothelial cells in ACI.

CCNG1, a member of the late cell cycle family, is one of the subtypes involved in cell cycle and proliferation and DNA damage repair by interacting with APP2A, MDM2, and other proteins. CCNG1 promotes cell proliferation and apoptosis.^38^^,^^39^ Hes1, Notch1, and VEGF are angiogenesis-related proteins, which play an important role in angiogenesis and development.^40^ The above angioproteins were selected to further analyze the expression of miRNA-122 and angiogenesis-related proteins at mRNA and protein levels, and it was found that in the miRNA-122 mimic transfection group, the expression of Hes1, Notch1, VEGF, and CCNG1 in mRNA and protein levels were significantly decreased after miRNA-122 was overexpressed, and the expression of related proteins was significantly increased after miRNA-122 inhibition. To predict the target of miRNA-122, the dual-luciferase reporter assay proved that CCNG1 was the direct target gene of miRNA-122. These results suggest that miRNA-122 may promote cell apoptosis and inhibit vascular endothelial cell proliferation and angiogenesis by targeting the CCNG1 channel to regulate apoptosis-related factors Bax, Caspase-3, Bcl-2, and angiogenic proteins Hes1, Notch1, VEGF, and CCNG1. Finally, it plays a role in ACI.

In conclusion, serum miRNA-122 is significantly increased in ACI. Combined with inflammatory factors CRP and IL-6, it has important clinical value in diagnosing the severity and prognosis of cerebral infarction and is expected to be a biomarker for diagnosing and predicting the progression and prognosis of ACI. Similarly, in vitro experiments showed that miRNA-122 plays a regulatory role in ACI by targeting CCNG1 to inhibit the proliferation of vascular endothelial cells, and promote cell apoptosis, and angiogenesis. Large-scale multi-center prospective studies are needed in the future to further confirm the clinical value and related regulatory mechanism of miRNA-122 in ACI.

## Authors' contributions

Xiaojuan Yu, Tian Zhang and Zeng-Zhen Wei performed the majority of experiments and wrote the manuscript. Bin Gu, Ting Guo, Wen-Juan Jiang, Yue-Qin Shen and Dong Wang performed the remaining experiments and statistically analyzed the data. Qian Wang and Jun Wang designed the present study. Wen-Juan Jiang and Yue-Qin Shen authenticated the raw data in this study. All authors read and approved the final manuscript.

## Funding

The present study was supported by Taizhou Science and Technology Support Plan (Social Development, grant n° SSF20210117) and The Scientific Research Fund of Taizhou People's Hospital (grant n°  ZL202015).

## Availability of data and materials

The datasets used and/or analyzed during the current study are available from the corresponding author upon reasonable request.

## Ethics approval and consent to participate

The Ethics Committee of Taizhou People's Hospital approved the study (approval n° KY 201813701), and all samples were treated anonymously in accordance with ethical and legal standards.

## Patient consent for publication

The Ethics Committee of Taizhou People's Hospital approved the study (approval n° KY 201813701). The patient consented to the publication of the paper.

## Conflicts of interest

The authors declare no conflicts of interest.
